# Correction: Fungi, bacteria and oomycota opportunistically isolated from the seagrass, *Zostera marina*

**DOI:** 10.1371/journal.pone.0251536

**Published:** 2021-05-06

**Authors:** Cassandra L. Ettinger, Jonathan A. Eisen

Figs 5 and 6 are missing black triangles associated with several of the clasped clades. The authors have provided corrected versions here.

**Fig 5:**
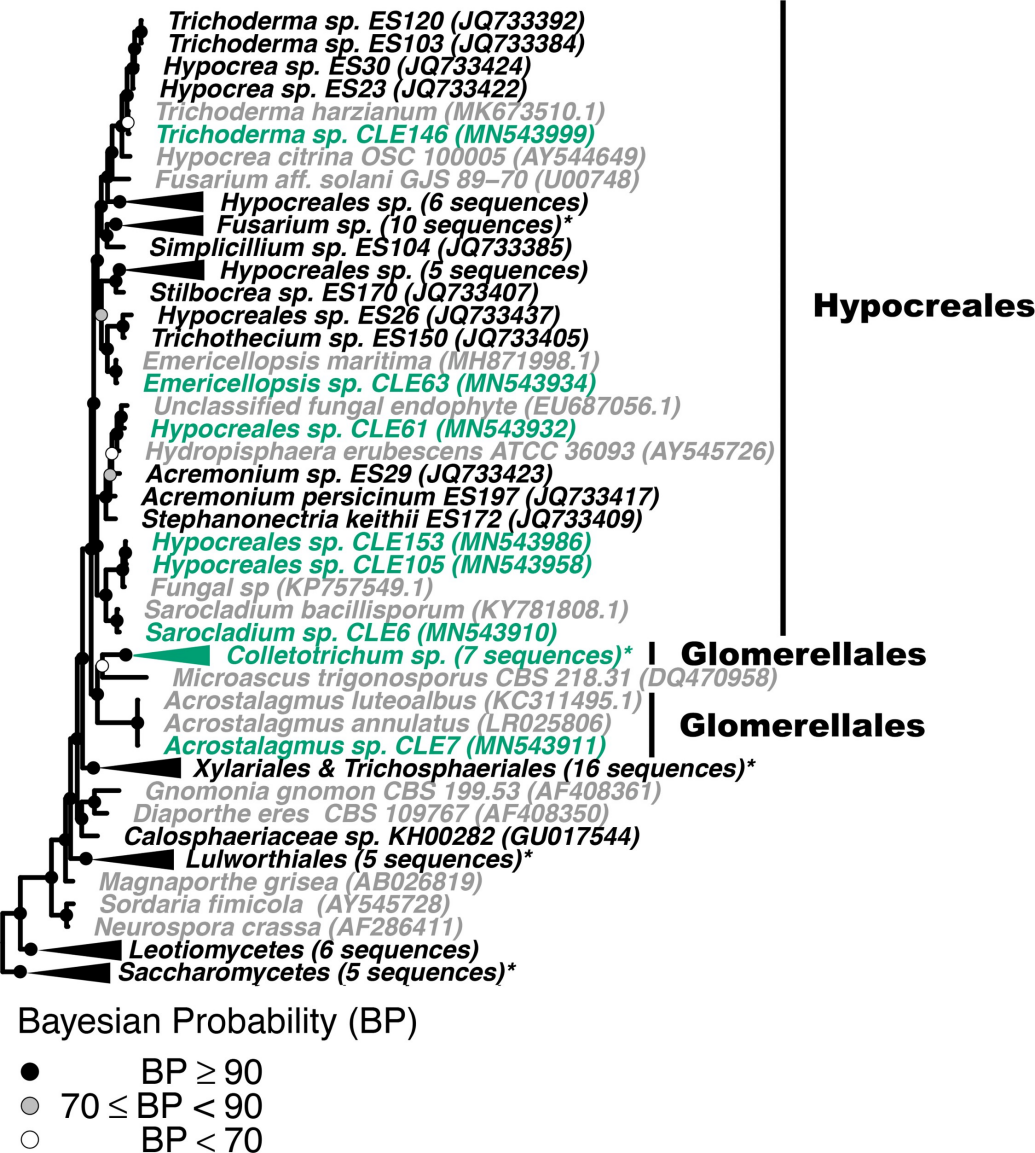
Phylogenetic placement of seagrass fungal isolates in the Sordariomycetes. A molecular phylogeny of 28S rRNA genes for isolates in the Sordariomycetes was constructed using Bayesian inference. This alignment was generated using MAFFT (v. 7.402) on the CIPRES Science Gateway web server, trimmed using trimAl (v.1.2) and a phylogenetic tree was inferred on the trimmed alignment with a GTR + I + G model using MrBayes (v. 3.2.2) [75–77, 81]. Displayed at each node as a circle in the tree are the Bayesian posterior probabilities (e.g. a black circle represents probabilities greater or equal to 90%, a grey circle represents probabilities greater or equal to 70%, a white circle represents probabilities less than 70%). The names of fungi isolated from *Z*. *marina* are shown in green, fungi isolated from other seagrass species are shown in black, and all other fungi are shown in grey. For visualization purposes, selected clades have been collapsed and the number of sequences within that clade is indicated. Collapsed clades are shown in green if the majority of sequences in the clade are from isolates associated with *Z*. *marina*, black if the majority of isolates are from other seagrass species, and grey otherwise. Clade names that are followed by an asterisk contain sequences from multiple sources. An expanded version of this phylogeny can be found in S9 Fig. The GenBank accession numbers of the sequences used to build this phylogeny can be found in Tables 1 and S2–S4.

**Fig 6:**
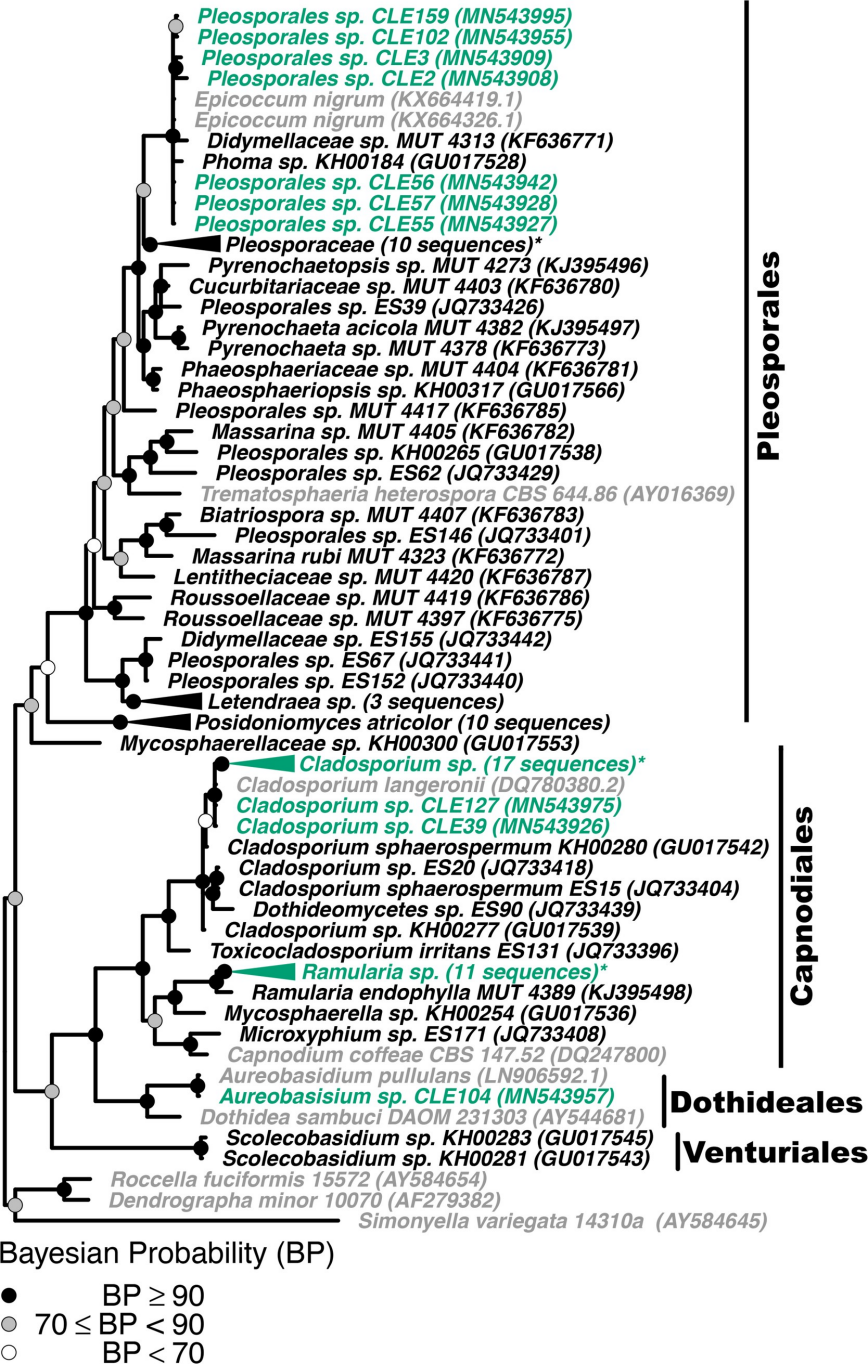
Phylogenetic placement of seagrass fungal isolates in the Dothideomycetes. A molecular phylogeny of 28S rRNA genes for isolates in the Dothideomycetes was constructed using Bayesian inference. This alignment was generated using MAFFT (v. 7.402) on the CIPRES Science Gateway web server, trimmed using trimAl (v.1.2) and a phylogenetic tree was inferred on the trimmed alignment with a GTR + I + G model using MrBayes (v. 3.2.2) [75–77, 81]. Displayed at each node as a circle in the tree are the Bayesian posterior probabilities (e.g. a black circle represents probabilities greater or equal to 90%, a grey circle represents probabilities greater or equal to 70%, a white circle represents probabilities less than 70%). The names of fungi isolated from *Z*. *marina* are shown in green, fungi isolated from other seagrass species are shown in black, and all other fungi are shown in grey. For visualization purposes, selected clades have been collapsed and the number of sequences within that clade is indicated. Collapsed clades are shown in green if the majority of sequences in the clade are from isolates associated with *Z*. *marina*, black if the majority of isolates are from other seagrass species, and grey otherwise. Clade names that are followed by an asterisk contain sequences from multiple sources. An expanded version of this phylogeny can be found in S10 Fig. The GenBank accession numbers of the sequences used to build this phylogeny can be found in Tables 1 and S2–S4.
