# Supplementary figures and images for: Fungi, bacteria and oomycota opportunistically isolated from the seagrass, Zostera marina
Source: PLoS One. 2020 Jul 22;15(7):e0236135. doi: 10.1371/journal.pone.0236135 (PMC7375540; doi:10.1371/journal.pone.0236135)

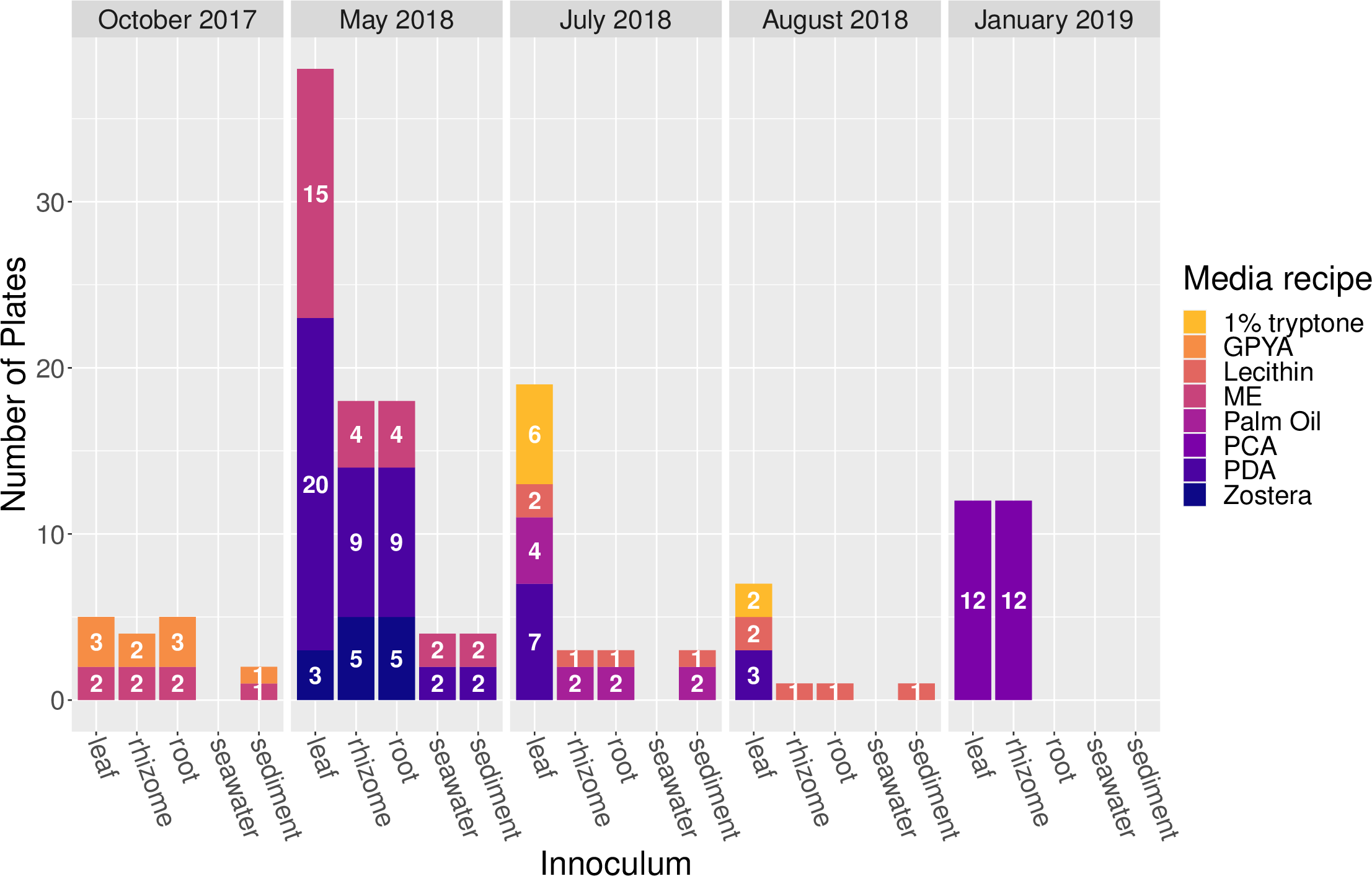

Supplement: S1 Fig — A histogram representing the number of inoculated plates faceted by collection trip (October 2017, May 2018, July 2018, August 2018, January 2019), grouped by inoculum source (leaf, root, rhizome, seawater, sediment) and colored by media recipe used for isolation. The media recipes used included 1% tryptone agar, potato dextrose agar (PDA), potato carrot agar (PCA), palm oil media, lecithin media, malt extract agar (MEA), glucose yeast peptone agar (GYPA), and Zostera marina agar (Zostera). The numbers included on each bar represent the count of plates inoculated for that media recipe with each inoculum source after each collection trip. For example, the first column shows the count of plates from sampling on October 2017 from leaf samples, with three plates on GPYA and two on ME. (TIF) [file pone.0236135.s001.tif]

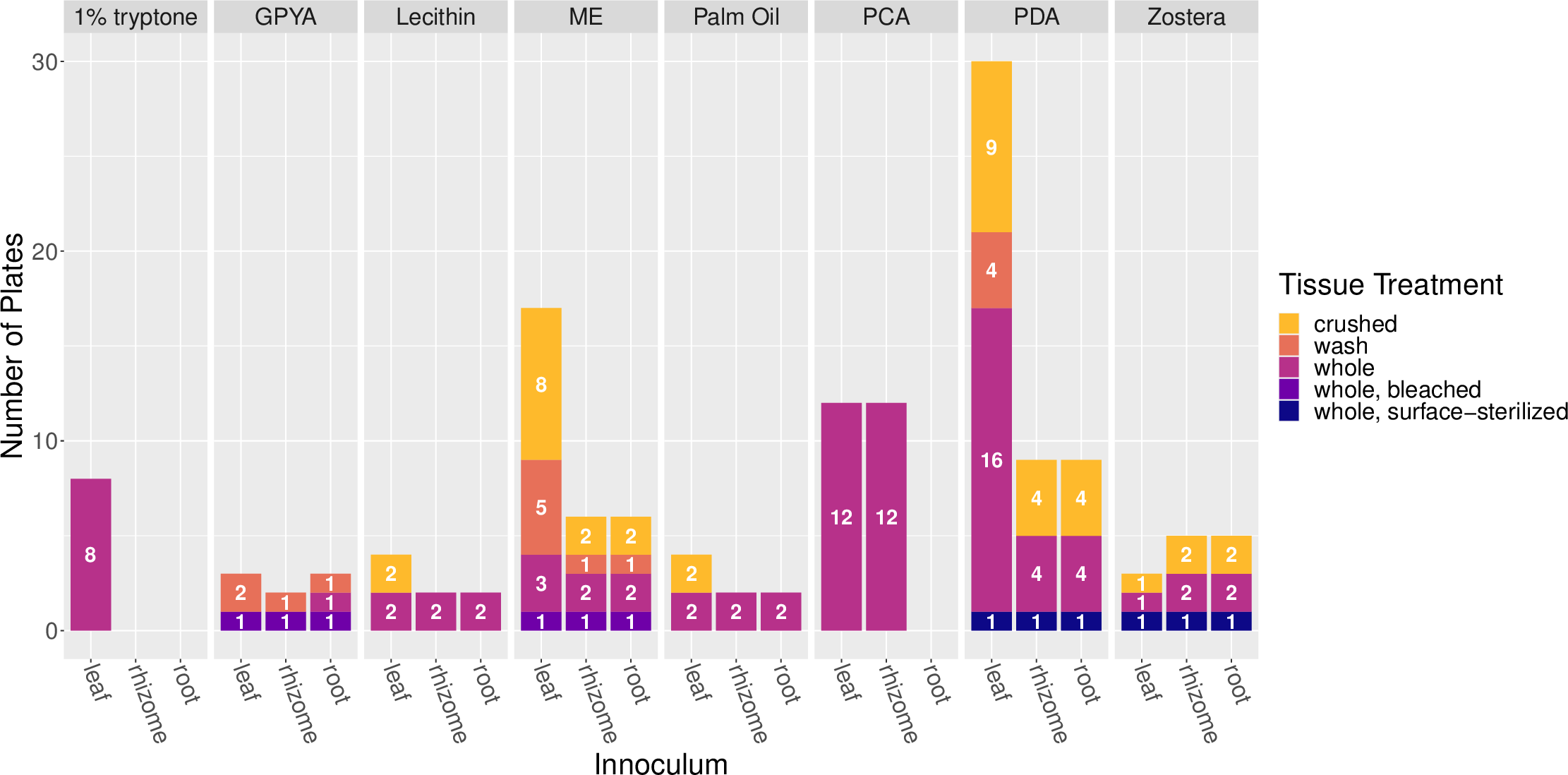

Supplement: S2 Fig — A histogram representing the number of inoculated plates faceted by media recipe, grouped by inoculum source (leaf, root, rhizome) and colored by tissue treatment (tissue wash, crushed tissue, rinsed whole tissue, bleached whole tissue, surface cleaned whole tissue) used for isolation. The media recipes used included 1% tryptone agar, potato dextrose agar (PDA), potato carrot agar (PCA), palm oil media, lecithin media, malt extract agar (MEA), glucose yeast peptone agar (GYPA), and Zostera marina agar (Zostera). The numbers included on each bar represent the count of plates inoculated for that media recipe with each inoculum source and tissue treatment combination. For example, the first column shows the count of plates inoculated on 1% tryptone from leaves, with eight plates inoculated with rinsed whole leaves. (TIF) [file pone.0236135.s002.tif]

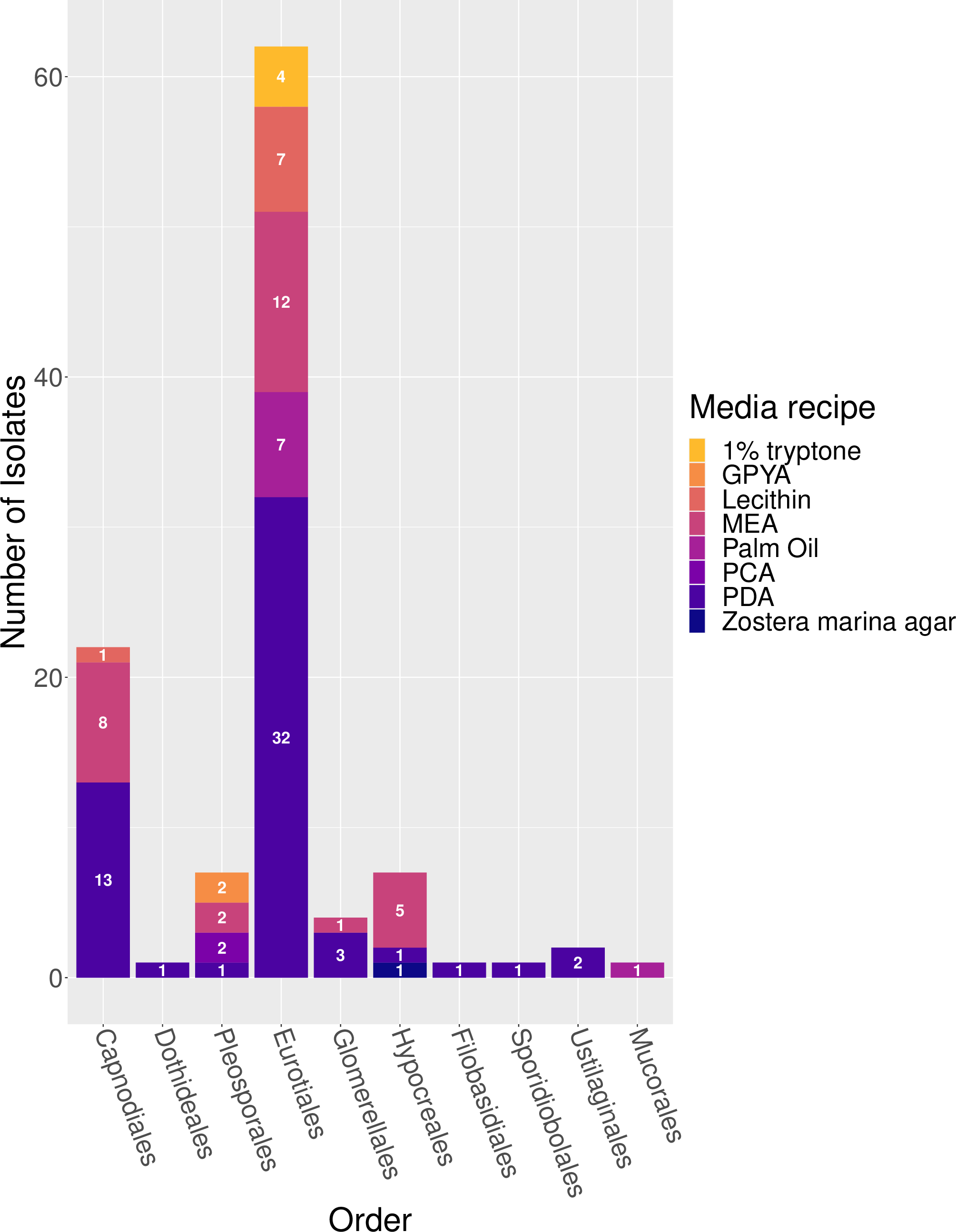

Supplement: S3 Fig — A histogram representing the number of fungal isolates grouped by order and colored by media recipe used for isolation. The media recipes used included 1% tryptone agar, potato dextrose agar (PDA), potato carrot agar (PCA), palm oil media, lecithin media, malt extract agar (MEA), and glucose yeast peptone agar (GYPA). The numbers included on each bar represent the count of isolates grown on each media recipe. (TIF) [file pone.0236135.s003.tif]

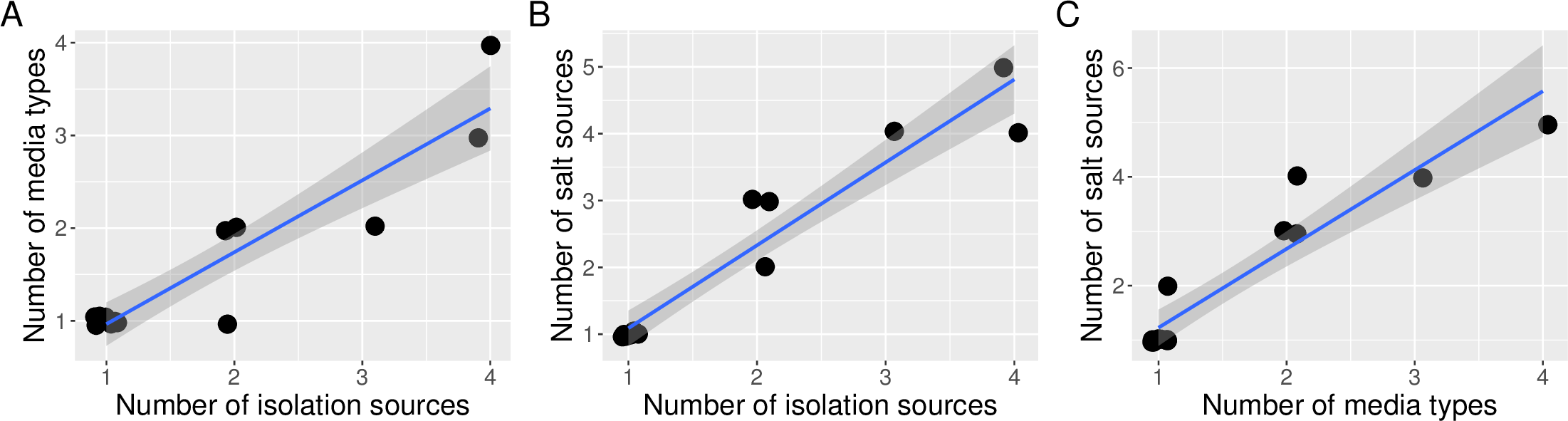

Supplement: S4 Fig — Scatter plots representing A) the relationship between the count of unique isolation sources (leaf, root, rhizome, sediment) and the count of unique media types (PDA, palm oil media, lecithin media, MEA), a fungal genus was isolated from (R2 = 0.86), B) the relationship between the count of unique isolation sources and the count of unique salt sources (no salt, varying amounts of instant ocean [8 g, 16 g, or 32 g], seawater) a fungal genus was isolated from (R2 = 0.92), and C) the relationship between the count of unique media types and the count of unique salt sources a fungal genus was isolated from (R2 = 0.87). (TIF) [file pone.0236135.s004.tif]

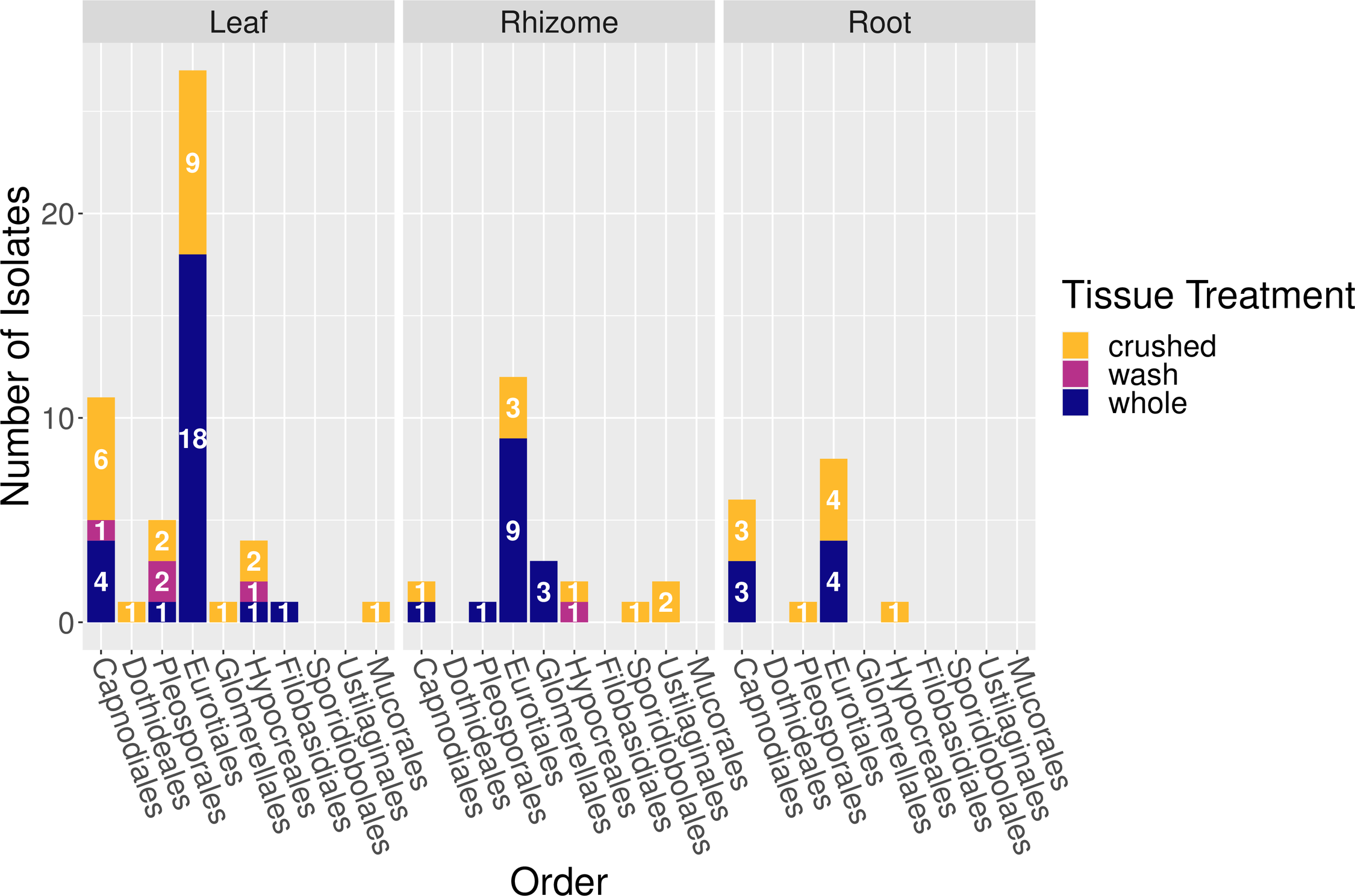

Supplement: S5 Fig — A histogram representing the number of fungal isolates faceted by inoculum source (leaf, root, rhizome), grouped by order and colored by tissue treatment (tissue wash, crushed tissue, rinsed whole tissue) used for isolation. The numbers included on each bar represent the count of isolates in each Order grown with each inoculum source and treatment combination. For example, the first column shows the count of isolates in the Capnodiales from leaf tissue, with six isolates obtained from crushed leaves, one from leaf washes, and four from rinsed whole leaves. (TIF) [file pone.0236135.s005.tif]

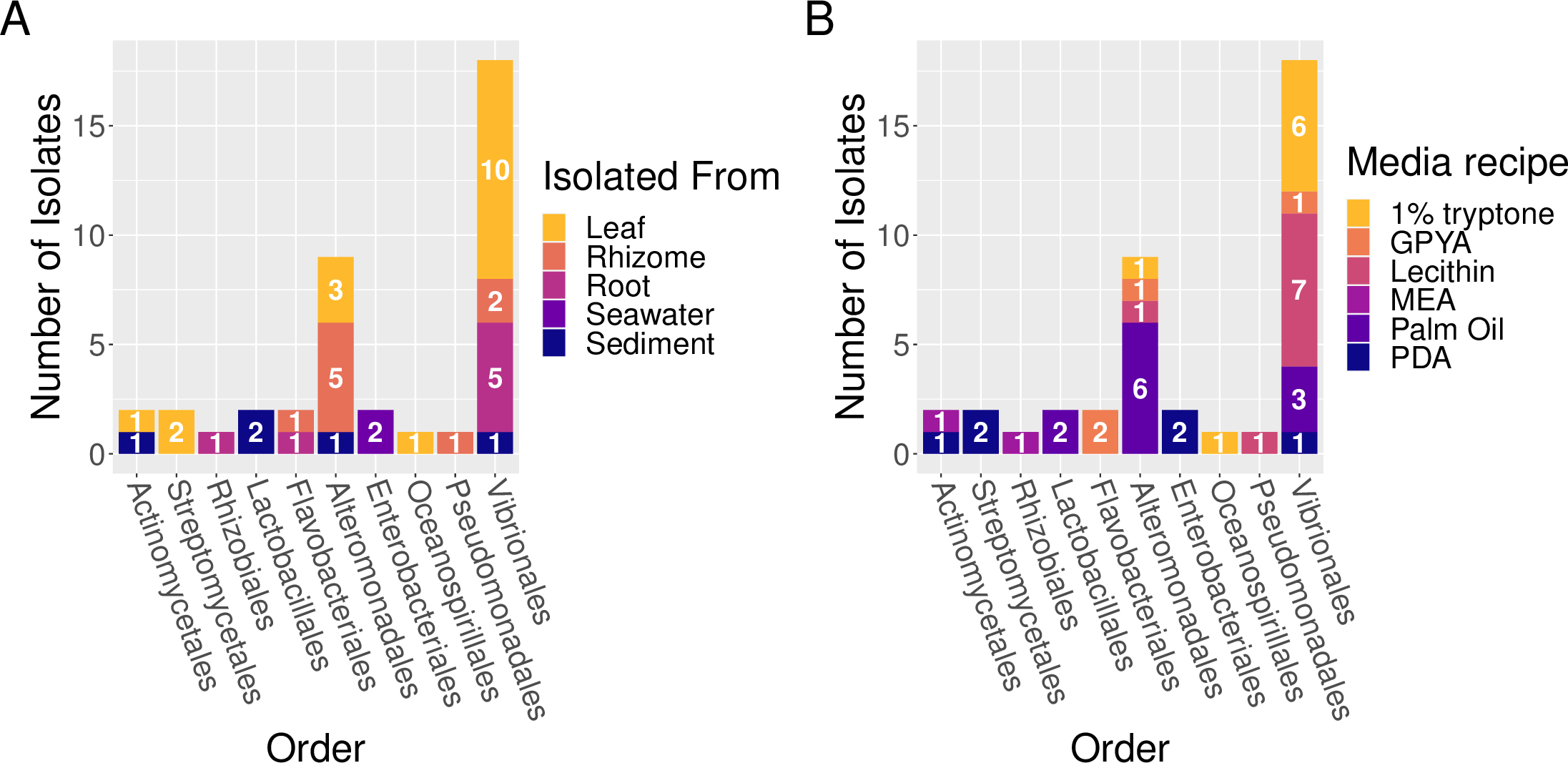

Supplement: S6 Fig — Histograms representing the number of bacterial isolates grouped by order and colored by isolation source (A) or media recipe (B). A) When colored by isolation source (leaf, rhizome, root, seawater or sediment), the numbers included on each bar represent the count of isolates obtained from that particular isolation source. B) When colored by media recipe used for isolation (1% tryptone agar, potato dextrose agar [PDA], palm oil media, lecithin media, malt extract agar [MEA], and glucose yeast peptone agar [GYPA]), the numbers included on each bar represent the count of isolates grown on each media recipe. (TIF) [file pone.0236135.s006.tif]

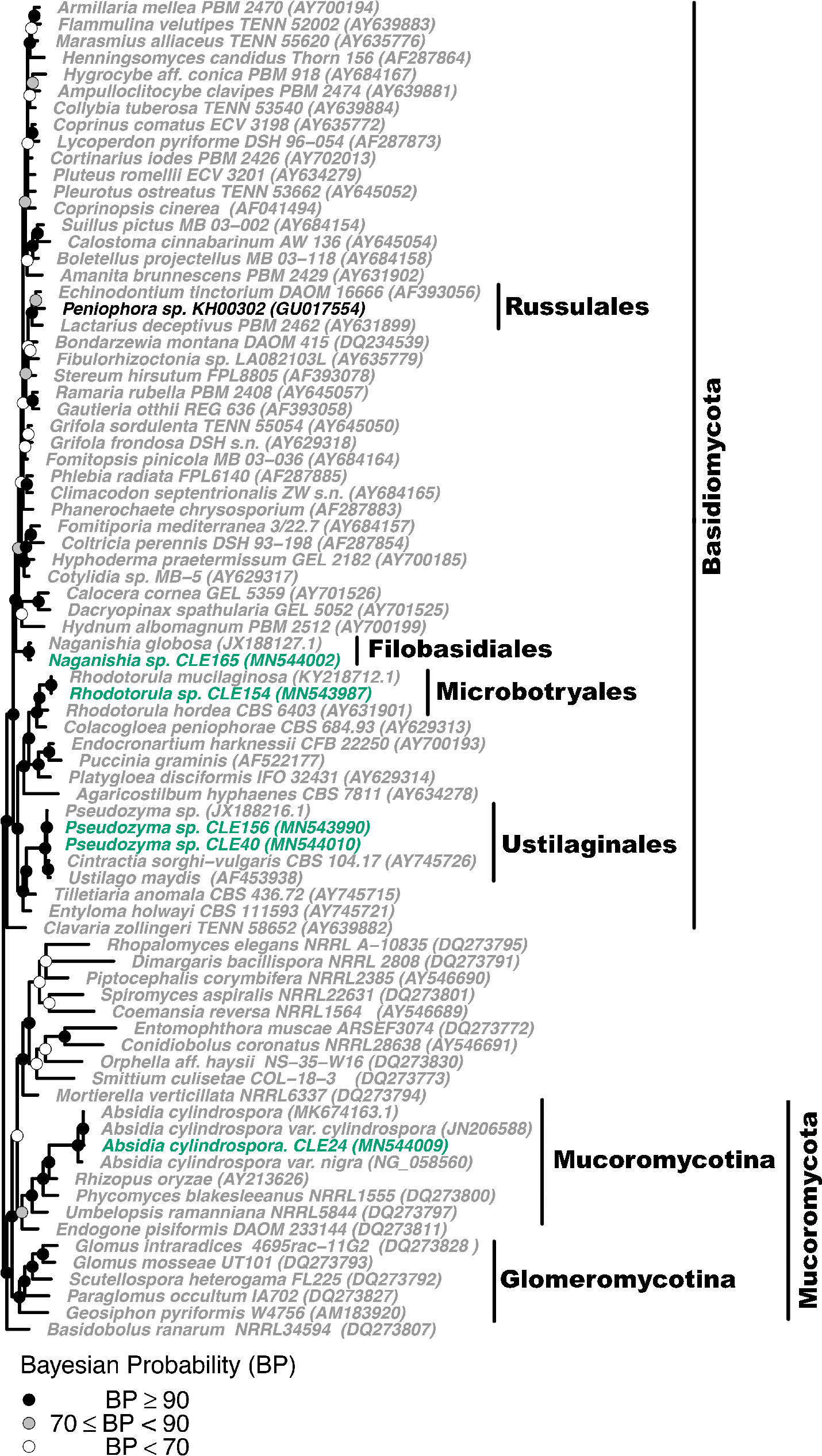

Supplement: S7 Fig — A molecular phylogeny of 28S rRNA genes for isolates in the Basidiomycota and Mucoromycota was constructed using Bayesian inference. This alignment was generated using MAFFT (v. 7.402) on the CIPRES Science Gateway web server, trimmed using trimAl (v.1.2) and a phylogenetic tree was inferred on the trimmed alignment with a GTR + I + G model using MrBayes (v. 3.2.2) [75–77,81]. Displayed at each node as a circle in the tree are the Bayesian posterior probabilities (e.g. a black circle represents probabilities greater or equal to 90%, a grey circle represents probabilities greater or equal to 70%, a white circle represents probabilities less than 70%). The names of fungi isolated from Z. marina are shown in green, fungi isolated from other seagrass species are shown in black, and all other fungi are shown in grey. For visualization purposes, selected clades have been collapsed and the number of sequences within that clade is indicated. The GenBank accession numbers of the sequences used to build this phylogeny can be found in Tables 1 and S2–S4. (TIF) [file pone.0236135.s007.tif]

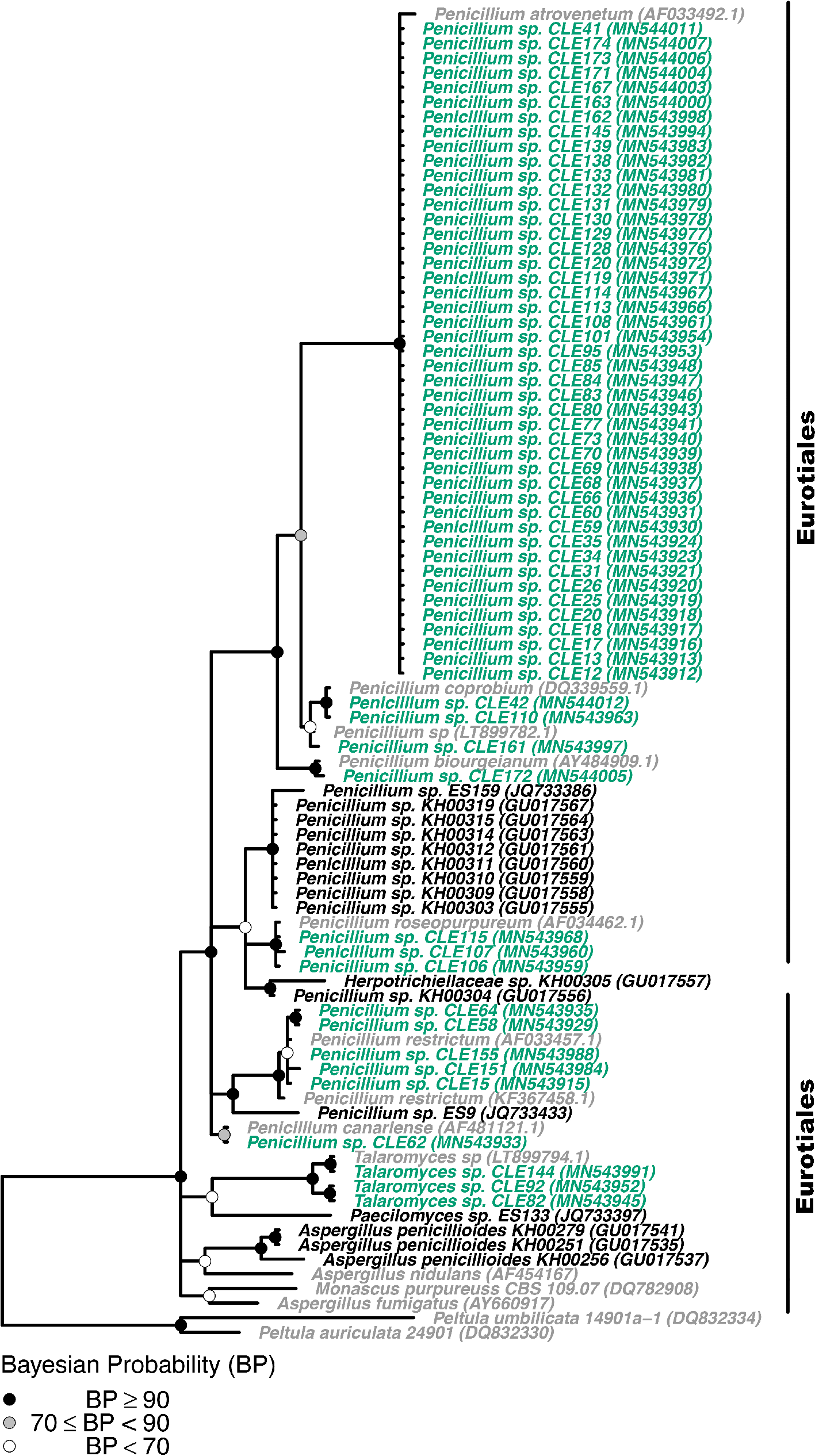

Supplement: S8 Fig — A molecular phylogeny of 28S rRNA genes for isolates in the Eurotiomycetes was constructed using Bayesian inference. This alignment was generated using MAFFT (v. 7.402) on the CIPRES Science Gateway web server, trimmed using trimAl (v.1.2) and a phylogenetic tree was inferred on the trimmed alignment with a GTR + I + G model using MrBayes (v. 3.2.2)[75–77,81]. Displayed at each node as a circle in the tree are the Bayesian posterior probabilities (e.g. a black circle represents probabilities greater or equal to 90%, a grey circle represents probabilities greater or equal to 70%, a white circle represents probabilities less than 70%). The names of fungi isolated from Z. marina are shown in green, fungi isolated from other seagrass species are shown in black, and all other fungi are shown in grey. The GenBank accession numbers of the sequences used to build this phylogeny can be found in Tables 1 and S2–S4. (TIF) [file pone.0236135.s008.tif]

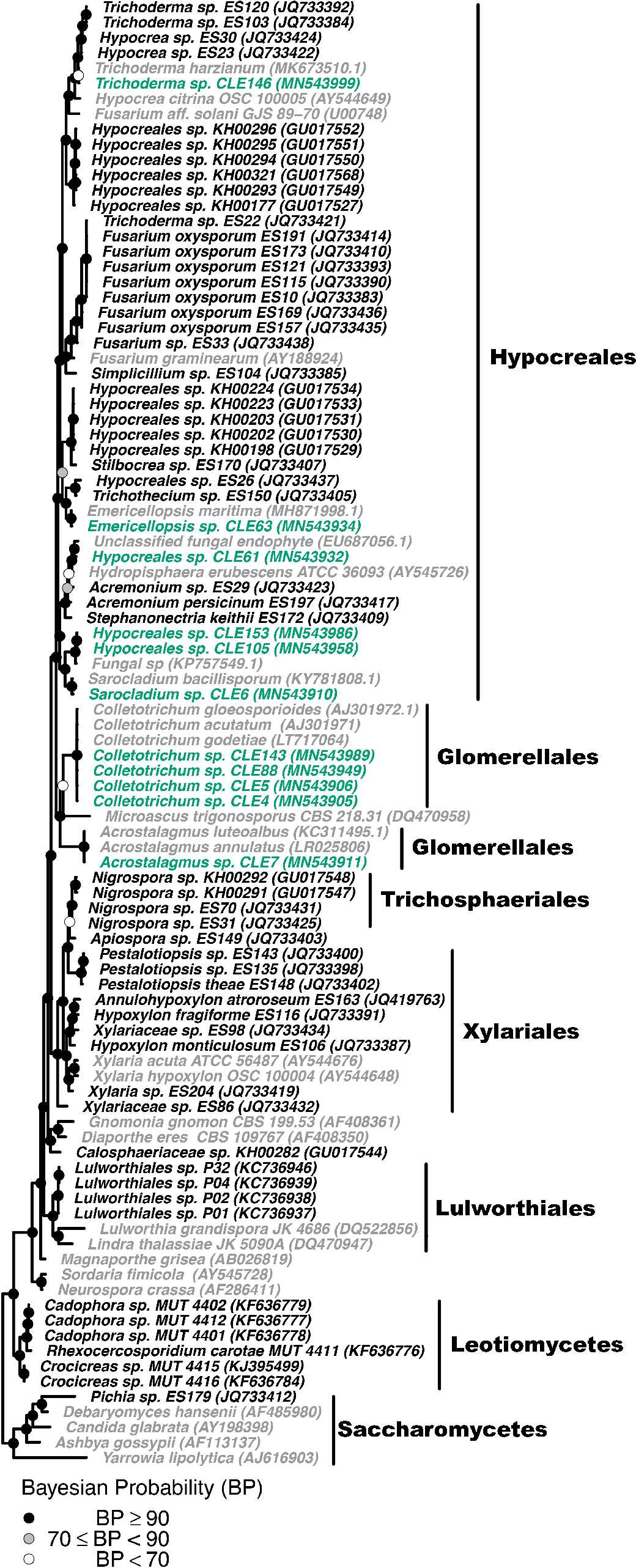

Supplement: S9 Fig — A molecular phylogeny of 28S rRNA genes for isolates in the Sordariomycetes was constructed using Bayesian inference. This alignment was generated using MAFFT (v. 7.402) on the CIPRES Science Gateway web server, trimmed using trimAl (v.1.2) and a phylogenetic tree was inferred on the trimmed alignment with a GTR + I + G model using MrBayes (v. 3.2.2)[75–77,81]. Displayed at each node as a circle in the tree are the Bayesian posterior probabilities (e.g. a black circle represents probabilities greater or equal to 90%, a grey circle represents probabilities greater or equal to 70%, a white circle represents probabilities less than 70%). The names of fungi isolated from Z. marina are shown in green, fungi isolated from other seagrass species are shown in black, and all other fungi are shown in grey. The GenBank accession numbers of the sequences used to build this phylogeny can be found in Tables 1 and S2–S4. (TIF) [file pone.0236135.s009.tif]

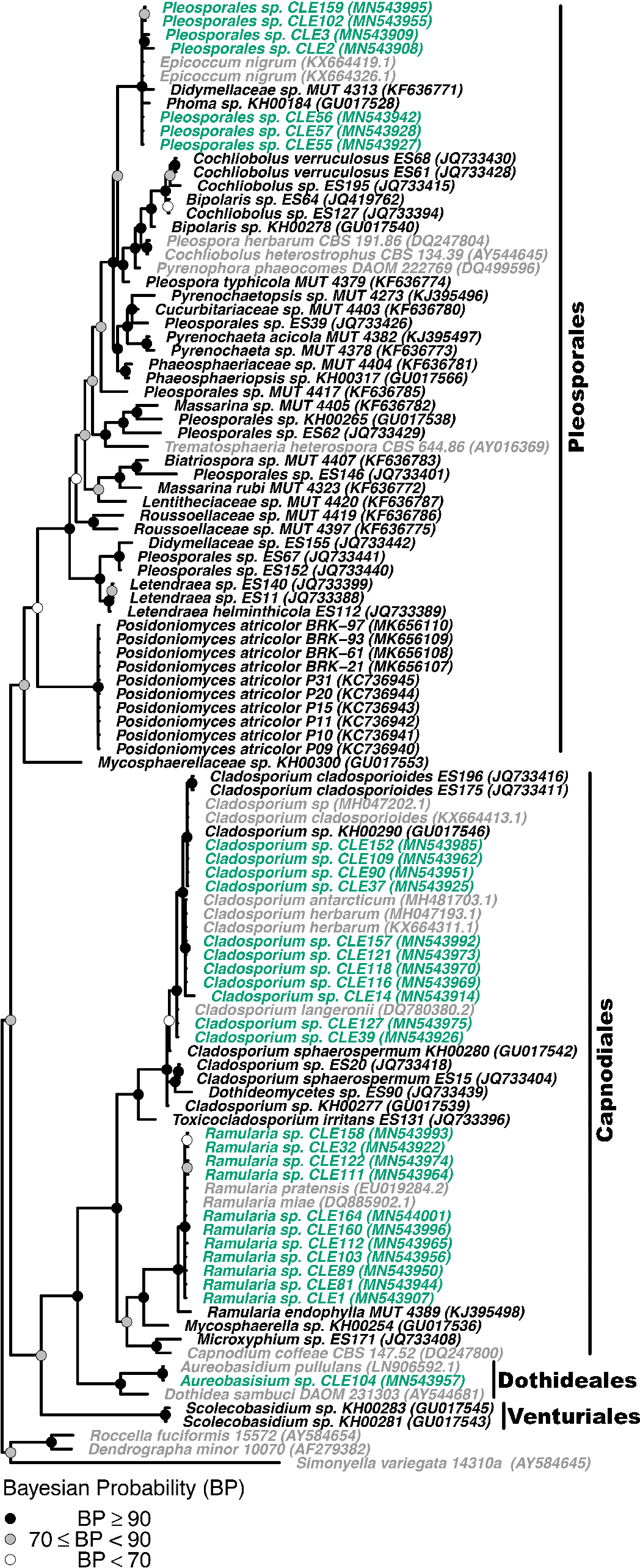

Supplement: S10 Fig — A molecular phylogeny of 28S rRNA genes for isolates in the Dothideomycetes was constructed using Bayesian inference. This alignment was generated using MAFFT (v. 7.402) on the CIPRES Science Gateway web server, trimmed using trimAl (v.1.2) and a phylogenetic tree was inferred on the trimmed alignment with a GTR + I + G model using MrBayes (v. 3.2.2)[75–77,81]. Displayed at each node as a circle in the tree are the Bayesian posterior probabilities (e.g. a black circle represents probabilities greater or equal to 90%, a grey circle represents probabilities greater or equal to 70%, a white circle represents probabilities less than 70%). The names of fungi isolated from Z. marina are shown in green, fungi isolated from other seagrass species are shown in black, and all other fungi are shown in grey. The GenBank accession numbers of the sequences used to build this phylogeny can be found in Tables 1 and S2–S4. (TIF) [file pone.0236135.s010.tif]

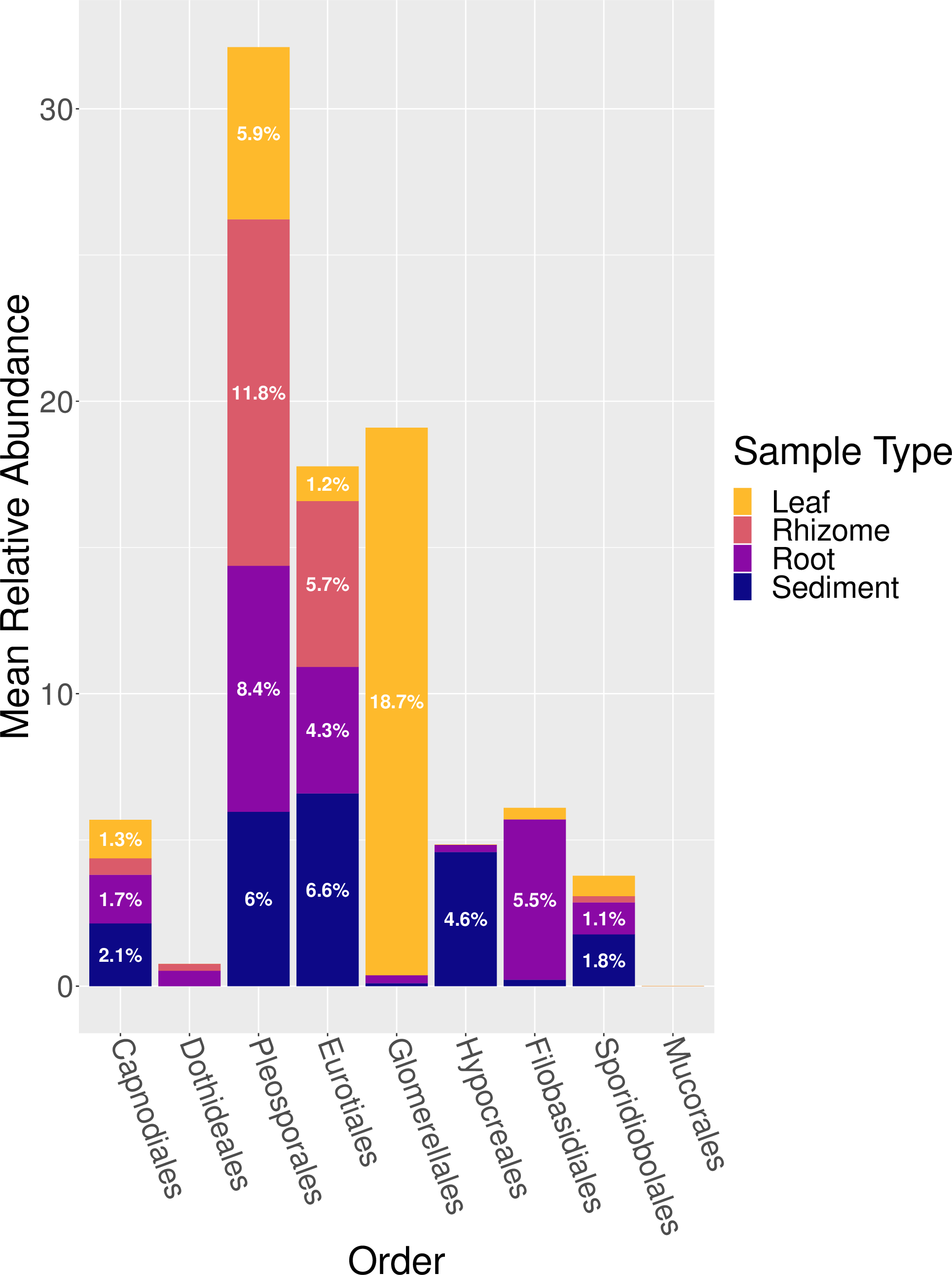

Supplement: S11 Fig — A histogram representing the mean relative abundance of amplicon sequence variants (ASVs) grouped by order and colored by sample type (leaf, rhizome, root, or sediment). The numbers included on each bar represent the mean relative abundance of the order detected on that particular sample type and only mean relative abundances greater than one percent are shown. (TIF) [file pone.0236135.s011.tif]
