# Supplementary material for: Fungi, bacteria and oomycota opportunistically isolated from the seagrass, Zostera marina
Source: PLoS One. 2020 Jul 22;15(7):e0236135. doi: 10.1371/journal.pone.0236135 (PMC7375540; doi:10.1371/journal.pone.0236135)
Supplement: Attachment — Submitted filename: Fungal Collection Response to Reviewers.docx [file pone.0236135.s017.docx]

Response to reviewers' comments:

**Reviewer #1: GENERAL COMMENTS**

**The authors present a culture-dependent study of fungal communities associated with Zostera marina resulting in a culture collection of fungi, some bacteria and a few oomycetes, which contributes to our still emerging understanding of marine fungi within seagrass ecosystems. Besides phenotypical characterization of obtained colonies, this research used sequencing and phylogenetic approaches to assess the reliability of the identities assigned to the different colonies, both within the context of seagrasses in general as well as in comparison with a previous culture-independent study of Z. marina. This original research included a variety of classic microbiology methods, high-standard analyses and conclusions that are supported by the data. However, the following general concerns should be considered for the review process to continue. Please also find below my specific comments and suggestions.**

**1. The rationale behind using three different markers (i.e. ITS-28S, 16S and 28S) should be explained. Why did the authors amplified these if they were pursuing a fungal collection?**

Initially, we did only perform PCR for the ITS-28S region. However, PCR failed for many isolates and so we then amplified the 16S rRNA gene (and the 28S rRNA gene) to identify these instead. We have now included text in the methods describing this rationale, L 271-275.

**2. Even though the methodological approaches are clearly explained, there are missing details that should be included in a revised version of the manuscript.**

We have attempted to address all areas pointed out to us by the reviewers in this revision and make point by point responses to reviewer comments below.

**3. Identification approaches get somehow mixed in the Results section due to the use of confusing/broad terminology (e.g. “molecular”), or just lack of specification (e.g. “Bacterial isolates were identified as belonging to…”). It is not clear whether the authors are talking about visual identifications, or those made based on taxonomic or phylogenetic analyses.**

No visual identifications were made. All identifications are taxonomic and based on molecular methods. Phylogenetic analysis was only performed for the fungal isolates and was congruent with the taxonomic identifications. We have removed some of the instances where we previously referred to “molecular methods” and added “taxonomically” in places.

**4. The discussion about patterns of distance decay is very interesting but its link with this study needs further explanation. The authors should consider expanding on the evidence they provide that supports a role of habitat specificity and/or dispersal efficiency at a local level.**

We agree that this is an interesting idea to explore, but we do not have the data (in this study) to support this further. We have rephrased the last sentence of this paragraph to better lead in to the results we do have of examples of habitat specificity (or lack thereof) in the following paragraphs.

**5. In general, table formatting should be improved.**

We apologize for the original table formatting. The tables were uploaded as an excel file to *bioRxiv*, which did not translate well during the pdf conversion to *PLoS One*. We have now re-formatted the tables and believe they are greatly improved.

**SPECIFIC COMMENTS**

**ABSTRACT:**

**Lines 60-62: It might be worth it to mention that Halophytophthora may also be just saprophytes.**

This has been added.

**Line 62: This study did not generate a culture collection of bacteria or oomycetes, as these microorganisms were not intendedly isolated, and therefore (as mentioned later in lines 502-504) are not representative of the true diversity of the culturable bacterial/oomycetal community associated with Z. marina.**

We have rephrased this statement only to refer to the fungal isolates.

**INTRODUCTION:**

**Line 75: The Introduction section may benefit from some background information about bacteria and oomycetes associated with seagrasses to support the discussion about these microorganisms, provided later provided in the manuscript (lines 501-521 and 523-532).**

We have added two paragraphs to the introduction to address this.

**Lines 90: “Blue carbon” is not an example of the preceding sentence, which indeed defines what blue carbon is. I suggest changing “e.g.” for “i.e.”.**

This has been fixed.

**Lines 111-113: Did the authors find any unidentified isolates? Based on this sentence, one would expect to see at least a proportion of fungi without taxonomic assignments. What about contrasting identities (i.e. different across identification methods)?**

We did have several fungal isolates for which were unable to assign taxonomy below the order level. We have added a few additional sentences to the results about these, L. 564-569. One reason we may not have as many unidentified isolates as might be expected, is that many of the unidentified amplicon sequences mentioned have closest matches to Chytridiomycota lineages and are unlikely to have been cultured using the methodology used here.

**METHODS:**

**Line 130: The authors state they collected “bulk leaf tissue”. Does this mean that tissue from all leaves from a single seagrass shoot was collected or there was a preference for a certain leaf rank?**

We have clarified this in the text. Bulk tissues were from multiple plants and no effort was made to standardize for a certain leaf rank, etc. Although only “green” leaf tissue was used for culturing.

**Lines 132-133: Is there a reason to keep samples in dark (and then incubate plates in dark too, lines 164-165)?**

This was done to be consistent with previous studies. We have added these references to the text.

**Line 135: Was there any attempt to cultivate anaerobic fungi, which may play important roles within the anoxic conditions typical of seagrass sediments? There are modifications of the plating technique that could help reduce Oxygen conditions (e.g. pour plate method).**

No attempt was made to cultivate anaerobic fungi. However, that is certainly an interesting future consideration! We have added some text to the discussion mentioning this limitation, L 681-683.

**Lines 175-179: An explanation of the criteria used to choose different extraction kits is missing. Why PowerSoil was not used for this particular subset of isolates?**

We tried out many available DNA extraction kits, but ultimately found the PowerSoil kit to be the best for DNA yield. However, samples that already had their DNA extracted were not re-extracted with the PowerSoil kit for cost reasons. We have added a line to the text about this L. 265-268.

**Lines 213-215: Were also the LSU, WARCUP and UNITE datasets used for oomycetes?**

Although all three databases are fungal-specific, we did run the sequences from oomycetes through all three databases. Only the LSU database had representative sequences from oomycetes though (suggested result of “unclassified oomycete”) and so we have now clarified that we ran the Fungal LSU database on the oomycetes in the methods.

**RESULTS:**

**Line 313: “A total of 108 fungal isolates were obtained” … From how many initial samples?**

We have now added a paragraph to the results section including additional information about the number of total plates inoculated, how many had observed microbial growth and how many resulted in the isolates reported here. L 438-464.

**Lines 315-317: “Combined” samples are not mentioned in the Methods section.**

We would like to thank the reviewer for pointing this out. There were no “combined” samples, these were data entry errors by undergraduate interns (e.g. “leaves and roots” was supposed to be entered as “leaves from base, near roots”, etc). We have corrected this throughout the text and the figures and double-checked all data-entries for accuracy.

**Lines 338-341: Confusing sentence. Suggest re-phrasing.**

We have rephrased this.

**Lines 345-346: An explanation of why this happens is required, since the use of antibiotics is specified in the Methods. The (close) phylogenetic relationship or other similarities between fungi and oomycetes should also be mentioned.**

We have added a sentence to the results explaining this, L. 516-520.

**Line 351: Where these bacterial isolates identified by sequencing, phylogenetic analysis or visual characterization of colonies? This should be clearly stated here and in similar sentences throughout the manuscript (e.g. line 360, etc.).**

All isolates were identified via sequencing. We have attempted to clarify this by adding “taxonomically” in a few places throughout the text.

**Lines 391-392: Where these two genera rare members of the microbiome?**

Detection is based on presence / absence and thus, these genera were not present at all, not even at low levels, in the sequencing data. We have added a sentence suggesting some reasons for their absence, L 600-602.

**DISCUSSION:**

**Lines 414-418: Missing a period in between these two sentences. Change “,” before “Members of the Sordariomycetes…”.**

This has been fixed.

**Lines 432-439: This paragraph might benefit from a brief discussion on anaerobic fungi and the limitations of the study in this regard. Please refer to my comment on line 135 below.**

We have added text to the discussion mentioning this limitation, L 681-683.

**Lines 449-451: Explain further. Which results suggest such a role?**

We have rephrased this sentence to better lead into the next two paragraphs on habitat specificity (or lack thereof).

**Lines 523-524: There are good examples of plant pathogenic oomycetes in the literature.**

We have now added several references to address this.

**Lines 528-521: Please correct the reference at the beginning of the sentence (it is missing the year) and delete the one at the end (not necessary).**

This has been fixed.

**FIGURES AND TABLES:**

**Figure 1: The legend should specify which plates correspond to the different taxonomical groups (i.e. bacteria, fungi or oomycetes).**

We have now added putative isolate taxonomies for each plate to the legend of Figure 1.

**Reviewer #2: This study aimed at characterizing the cultivable mycobiota of Zostera marina from a sampling site in Bodega Bay, California. The authors also report some bacteria that were obtained in spite of the antibiotics used against their growth and two conspecific oomycetes obtained by chance.**

**Q1: Does the title of your MS fully reflect the merit of your study? Does an a priori biased bacterial spectrum resistant to antibiotics + two accidentally obtained conspecific oomycetes (typically parasites) warrant especially the first part of the title, which reads “All small things considered”? Not least, there are many other “small things” associated with seagrass surfaces (Bryozoa, Cyanobacteria, Foraminifera, microalgae, etc.), so I would suggest choosing a more conservative/informative title for your manuscript.**

We intended this to be word-play on the NPR program “All things considered”, but we agree with the reviewer that not all small things are considered and thus, it may be interpreted as misleading. We have changed the title to “Fungi, bacteria and oomycota opportunistically isolated from the seagrass, *Zostera marina”* to address this.

**The authors “opportunistically” collected “individual” plants of this seagrass during “several sampling trips between summer 2017 and summer 2018”. They also collected “bulk leaf tissue” and seawater.**

**Q2: It seems like this MS represents random results of some (actually, how many?) random (“opportunistically”) collections during one year rather than a targeted study aimed, e.g., at the seasonal effect on the respective mycobiota or the effect of different plant tissues. What is then the reason of publishing these apparently random data in the current form? If the aim was to provide reference material for further studies, i.e., in this case nrDNA sequences, I suggest their deposition in the INSD would do the same job. If the aim was to establish some reference collection of fungi, then these must be first somehow characterized taxonomically (=at the species level) and deposited in a way that they are available to other researchers – even this, however, does not need to be connected with this type of publication. If the aim was to compare the cultivable community with your previous NGS data – then I suggest the authors use at least the same way of sampling (i.e., “at three timepoints 2 weeks apart spanning July to August”), otherwise any such comparisons make little sense to me.**

We accept that what is reported here is not the result of a simple, clean project design and that some aspects of the work could be described as opportunistic (we disagree with the suggestion that this is random). Certainly additional time points, or different sampling design or culturing protocols or other alternatives could be done in future studies and could lead to more novel or significant results. But we believe this manuscript is fully within the scope of *PLoS One* and meets the seven criteria outlined on the *PLoS One* site. We have tried to be incredibly careful to not overstate our results and conclusions and believe that the results are technically sound and of potential value to the community as currently described.

**Q3: Considering the sampling protocol, I am not sure whether the authors were actually able to distinguish “individual” Z. marina plants. If yes: why did you collect “bulk leaf tissue”? How many individual plants were actually collected during each sampling, how many in total? These are important data that may help to understand the diversity and abundance of the mycobiota per one such “individual” plant.**

We never set out to characterize individual plant mycobiota, just any cultivable fungi associated with *Z. marina*. We used the cores as a way to be able to (1) obtain closely associated sediment and seawater, (2) they are easier to transport back to the lab while maintaining a ‘natural’ environment (e.g. the *Z. marina* remains submerged in seawater in the core and roots / rhizomes, etc, are still in the sediment). We also collected bulk tissues both for culturing efforts and also to use in media recipes (we tried both boiling *Z. marina* leaf tissues and dehydrating leaves for inclusion). We have expanded the methods here to help clarify this.

**For isolation of the mycobionts, the authors used several cultivation media. For obvious reasons this is a good idea, despite that the available seagrass studies indicate only a little effect of this variable. However, it is not easy to evaluate the different media effect in this study because some important information is missing.**

**Q4: Did you always use the whole range of these media, with the same number of replicates? How many replicates (per seagrass individual, tissue, sediment, seawater, etc.) did you actually establish per sampling?**

In short, no. This paper actually describes the work of five different undergraduate culturing efforts and each involved a different amount of replication across media types / tissues. We have now added a paragraph to the results section including additional information about the number of total plates inoculated, how many had observed microbial growth and how many resulted in the isolates reported here, L 438-464. We have also added some supplemental figures to help relay some of this information (e.g. number replicates per collection date) as well.

**The authors did not surface-sterilize the investigated tissues (rinsing them in autoclaved water is definitely not enough to get rid of the plethora of surface-dwelling organisms, including their resting stadia). This approach prevents recognition of organisms living inside these tissues (intra- or intercellularly) from the only randomly associated ones.**

**Q5: Was there any particular reason for omitting surface sterilization? One can ask a simple question, is the mycobiota detected in your study indeed somehow specific to Z. marina or is it just a random spectrum that could be detected +- on any other submerged surface at your sampling site?**

**The isolation experiment lasted only 4 weeks which is definitely a very short period as many marine fungi are notoriously slow growing (some need many months to start to emerge from the seagrass tissues). I understand it may be difficult to wait one year for the results but your sampling actually spanned over one year so at least the first isolations could have been kept for a longer time (perhaps at least 2-3 months).**

That is an interesting question. We actually did do some surface sterilization, and have expanded the methods to reflect this. Unfortunately, none of our final isolates came from the surface sterilized tissues. Additionally, we were interested in any fungal isolates that could be obtained from the seagrass ecosystem, both those tightly associated and loosely associated.

We actually kept most plates > 3 months at room temperature, but not all plates were kept this long and we were conservative in the text (parent plates were kept a minimum of 4 weeks). We have now changed this to be a range of 4-12 weeks in the text. We actually kept all parent plates and subcultured plates at 4°C while still working to isolate each microbe. All plates associated with an organism were only tossed after DNA extraction and cryopreservation of each organism.

**The authors obtained 108 fungal isolates but again, some important info is missing to fully understand the meaning of this number and its context.**

**Q6: How many replicates did not yield any isolate? How many isolates did you obtain in total, how many of them were non-axenic? How many did you attempt to identify, in how many cases you failed (low quality signal, chimeric sequences – perhaps a sign of non-axenic isolates)? Indeed, it seems that not all isolates were eventually sequenced (L. 171: “from the majority of isolates”) so one could ask why?**

The line referred to was supposed only to indicate that different DNA extraction kits were used for some isolates vs. the majority. We have edited the language to make that more clear. We have also added an additional paragraph to the results section (L 438-464) describing how many total isolates were generated, how many of those were successfully identified and from how many parent plates, etc.

**One of the ways how to distinguish randomly associated surface dwellers and spores from, e.g., true endophytes would be to compare results from the plated tissue segments and the wash liquid (L. 135-147 in Materials and Methods). The latter is, however, not stated in Results.**

**Q7: Why there are no results mentioned for the wash liquid? It is hard to believe that the wash liquid would have produced no fungi.**

Since we were interested in both loosely associated and tightly associated fungi, and because none of the surface-cleaned or bleached tissues resulted in any fungal isolates here, we had lumped together isolates from washes and rinsed tissues as just being isolated from “leaf”. We also note, that only a handful of isolates were actually obtained from washes (5 fungal isolates and 5 bacterial isolates), and none of the isolates from the washes were *Penicillium* sp. We have now added several new columns to S1 Table on whether an isolate was obtained from wash or rinsed tissue, as well as if it was from bulk plant tissue or a core, and the date the inoculum for each plate was collected.

**Q8: The Result section includes data for “combined leaf and root tissues” and “combined root and rhizome tissues” – what do these “combined” treatments mean/represent? They are not mentioned in Materials and Methods.**

We would like to thank the reviewer for pointing this out. There were no “combined” samples, these were data entry errors by undergraduate interns (e.g. “leaves and roots” was supposed to be entered as “leaves from base, near roots”, etc). We have corrected this throughout the text and the figures and double-checked all data-entries for accuracy.

**Most of the isolates obtained (n=59) belonged to Penicillium sp., which are ubiquitous fungi occurring practically everywhere especially in the form of spores – this is probably the answer to the question the authors asked themselves (L. 550-551): “For our study here we could ask - are many of the proposed habitat generalists actively growing in the seagrass ecosystem or merely passing through as spores?”. Does it make sense to attribute these specifically to Z. marina?**

It is certainly true that *Penicillium* sp. can be isolated from a large number of samples and environments. However, we are not trying to attribute any of the organisms we cultured specifically to *Z. marina.* We are reporting what we were able to culture. Some of these isolates may represent taxa with tight associations with *Z. marina* and some may not. In addition, even though *Penicillium* sp. overall can be isolated from diverse sources, it is likely that there is significant biogeography in terms of which varieties are found in which places. Therefore, we believe it is important to report on and examine the *Penicillium* isolates we have been able to obtain here.

**Q9: What does it mean when you state that you did not “attempt to control for effort or isolation frequency in this study”? Please be specific.**

We have rephrased. Simply, we did not control for replication across tissues / media types. This is the result of the culturing efforts reported on in this paper actually being the results of five distinct undergraduate culture projects.

**There was a little overlap between the fungi isolated here and in other studies and one of the explanations was that this could be “the result of the limits of culture-dependent studies generally or the use of different media recipes and methods for isolating fungi in other studies.”**

**Q10: What are the “general limits of culture-dependent studies” that could have caused these differences? Is there any “general limit” in you study that differs from other studies? I cannot find any particular…**

It is now well known that culture-dependent studies are not representative of the true diversity of microbiota associated with an environment, host, etc. All the discussed culture-dependent studies are biased by this, and that is all we meant here. We have rephrased to remove confusion.

**Q11: Ad different media recipes, here is what you state in L. 311-313: “…we used a variety of media types including several which had been used previously to isolate fungi from seagrasses (e.g. PDA (Sakayaroj et al., 2010; Vohník et al., 2016; Supaphon et al., 2017), GPYA (Panno et al., 2013), MEA (Torta et al., 2015)).”. Did you detect any similarities at least for these media (PDA, GPYA, MEA)? What other methods of isolation did the other studies use? As far as I know they were actually very similar to your methodology…**

Media recipes are provided in the Methods section, L 222-237. The fungal sequences from Sakayaroj et al. (2010), Vohník et al. (2016), and Supaphon et al. (2017) are all included in the phylogenies in this study. So yes, *Penicillium* sp*.,Trichoderma* sp*.,* *Cladosporium* sp*.* and *Ramularia* sp*.* are all taxa that had been previously isolated from some of these (and other studies). Our results are also consistent with many studies that did not have LSU sequence data available (including Panno et al, 2013).

The biggest differences in methodology are (1) the use of a different seagrass species from a different geographic area, and (2) the broad approach taken here vs. a focus on only one tissue type (e.g. only on roots/rhizomes, or only on leaves) which was the case for many of the other studies with available LSU sequence data.

**The authors wrote that “The fungal taxa that did have close relatives that were associated with other species [in other studies] included Penicillium sp….”/“Penicillium sp. and Cladosporium sp. were some of the only fungi in this study which were found to have close relatives associated with different seagrass species”. Indeed, Penicillium is everywhere and for example in culture-based studies of endophytes, these fast growing sporulating isolates are typically discarded as contaminants. At the same time, the authors note that “Penicillium sp., Cladosporium sp. and Ramularia sp. are drivers of the positive relationship observed previously between the number of tissue types and number of media types a fungal genus was isolated from”…**

**The main result of this study seems to be the establishment of the collection of the 108 fungal isolates “expanding understanding of the diversity of Z. marina associated fungi”. The collection actually comprises ca. 25 different fungal “species”, ca. 7 of these (more than 70 isolates) are ubiquitous sporulating fungi and 11 are represented by only one isolate – I suggest the authors use a more conservative/modest expression than “to expand”.**

We have changed the terminology from “expands” to “adds to” in the abstract and conclusion.

**Minor comments:**

**Line 48: endophytes are typically defined as symptomless plant endosymbionts and while I acknowledge that they may have secondary beneficial effects on their hosts, they can be to the same extent harmful, e.g., when environmental conditions change, and typically function along the so-called mutualism-parasitism continuum. An easy way how to solve this might be deleting “beneficial” and perhaps also listing mycorrhizal fungi (which are not endophytes, e.g., see Wilson 1995 Oikos 73:274–276)**

We have removed ‘beneficial’ here.

**L. 54: Penicillium sp., etc. – “sp.” not in italics**

This has been fixed throughout the manuscript.

**L. 58-60: Please refrain from the remarks about “interesting secondary metabolites” and “nitrogen cycling in the seagrass ecosystems” – these claims cannot be supported by any results presented in your manuscript.**

This has been removed.

**L. 81: Comments as for L. 48**

We have removed ‘beneficial’ here.

**L. 135: Please state, at least approximately, the time interval between the sample collection and the tissue plating (the length of this interval may significantly affect the obtained symbiont spectra)**

This has been added.

**L. 136-7: Please detail the rinsing of tissues in autoclaved water – how many washes, for how much time, etc. In case someone wants to compare your protocol with other studies…**

This has been added.

**L. 140: Perhaps use 1 ml instead**

This has been changed.

**L. 166-7: This implies that some of the obtained isolates were non-axenic; how did you recognize these and how/on what basis could you be “confident” that the final cultures were axenic?**

We have added more detail to the methods to describe this, L. 244-253.

**L. 314: Fig. 1 is nice but redundant and since it is practically the same as Fig. 1 in Amend et al. 2019 I suggest omitting it**

We disagree that this figure is redundant. It provides a visual presentation of the diversity of the cultures. Yes, it is structured in the same way as the figure from Amend but that figure showed included isolates from a marine sponge. We do not believe that the fact that Amend et al. also showed plates with cultures should lead to this figure being removed from this paper. We note - similar figures have been used in other papers including Ogórek et al. <https://doi.org/10.3390/d11100191>, Hamzah et al. <https://doi.org/10.3389/fmicb.2018.01707> Doi et al <http://dx.doi.org/10.1590/1676-0611-bn-2017-0416>, Le et al. <https://doi.org/10.1038/s41598-019-52481-2> Sobral et al. <http://dx.doi.org/10.1590/0001-3765201720160903> and many others. This is a common type of figure to include in “fungal culture diversity” papers.

**L. 359: Z. marina not maraina**

This has been fixed.

**L. 426: Mediterranean not mediterranean**

This has been fixed.

**L. 427-428: “associated with changes in root hair morphology” – not true, please read carefully the paper by Borovec and Vohnik 2018**

We have changed “morphology” to “development” to address this.

**References must be doublechecked – for example, there is no journal for Rodriguez 2008 (L. 730), L. 788 Kolařík not Kolarˇík, etc.**

Rodriguez 2008 is a master’s thesis, which was accidentally omitted based on the reference manager we were using. We have now updated the format to be in line with *PLoS* guidelines and have double-checked all the references.

**Table 1: The column “Genus detected in ITS amplicon data” has a very little information value; for example, Penicillium contains more than 300 species with varying biology so detecting “Penicillium” in both data sets tells nearly nothing…**

We agree that the significance of detecting Penicillium in both data sets is different from cases where one detects taxa that have very narrow phenotypic variation among isolates. However, we still believe this is important to report on. We have added text to the table legend to address this, L 1588-1590.

**Figure 4: The 45 Penicillium isolates seem to be conspecific – does it make sense to include all the 45 (practically indentical) sequences in the tree? The same holds true for other non-singleton isolates (perhaps better to establish some representative MOTUs?) and also for reference isolates from other studies (the trees are very large – one representative sequence would be enough).**

We have now collapsed clades of closely related sequences in Figures 4, 5 and 6.

**Reviewer #3: The manuscript by Ettinger and Eisen describes a non-targeted cultivation approach to isolate fungi from Zostera marina. The authors identify the strains using standard techniques and compare the data to their previously published ITS amplicon data. As by-catch, some bacteria and Oomycetes were cultured. The manuscript is straightforward albeit without clear directions or hypotheses that are being tested. Large parts of the data presentation (tables and figures) do not have sufficient quality for publication in their present form.**

**Comments:**

**1) Tables 1 and 2 are not in a form that is suitable for publication in a paper. I cant event read some of the column headers. Also, how were these calls made? What were the sequence identity cutoffs, etc. What is a ‘Molecular ID’?**

We apologize for the original table formatting. The tables were uploaded as an excel file to *bioRxiv*, which did not translate well during the pdf conversion to *PLoS One*. We have now re-formatted the tables in Word which should greatly improve this.

We have changed the column name from “Molecular ID” to “Putative taxonomy” to help clarify. Taxonomic assignments of sequences were obtained by comparing the best results (or “top match”) across three methods (NCBI, RDP, Silva ACT) to obtain a consensus assignment. For the fungal sequences, these assignments were confirmed phylogenetically. For the bacteria, all isolates were assigned the same genus by all three methods used.

**2) Tables 2/3: Top BLAST match. I think the authors can do much better to provide useful information on the ID of the bacteria. E.g., sequence similarity to the most closely related described species, not to a genbank entry with potential dubious history**

We have updated Table 2 to be based on top matches to the NCBI BLAST curated 16S ribosomal RNA sequence database for bacteria. We have left Table 3 as is. There are no sequences in RefSeq for any close relatives of this oomycete group and the top matches from BLAST to nr/nt are to an isolate considered the holotype for that species from the Westerdijk Fungal Biodiversity Institute.

**3) Figure 1: While this looks pretty, it is hardly scientific. The authors should indicate the strain names and why this subset of strains is shown here**

We have now added putative isolate taxonomies for each plate to the legend of Figure 1. We have also added a line to the figure legend stating that “Depicted plates were arbitrarily chosen to depict the morphological diversity of the isolates cultured in this study.”

**4) Figure 4,5,6: Isolates that are identical/nearly identical should get clustered**

We have now collapsed clades of closely related sequences in Figures 4, 5 and 6.

**5) Fig. 7: should show which genera were detected by amplicon sequencing or isolation only. Also, again more detail on how these calls were made is necessary. E.g. are the amplicons 100% matches to the isolates? Same applies to the 'yes' or 'no' for detection of the respective 'genera' in the amplicon data**

Figure 7 now differentiates which method genera were detected when only detected by one method. We only compared taxonomic calls between the isolate and amplicon sequencing dataset as described in the methods, L. 410-427.

**6) Line 908 and Figure S2: It’s one genus, genera is plural…**

We have fixed this in Figure 7 and Figure S2.

**7) L. 220: analyzed**

We believe that ‘analysis’ is the correct word in this case, as the data was imported into R for both visualization and analysis.

**8) ‘sp.’ like in Penicillium sp. is not in italics as it’s not a species name. Please correct throughout the paper**

This has been fixed throughout the manuscript.
